# Supplementary material for: Continual deep reinforcement learning with task-agnostic policy distillation
Source: Sci Rep. 2024 Dec 30;14:31661. doi: 10.1038/s41598-024-80774-8 (PMC11685974; doi:10.1038/s41598-024-80774-8)
Supplement: Supplementary file 1 — Supplementary Information. [file 41598_2024_80774_MOESM1_ESM.pdf]

Appendix A Progress and Compress Phases

Progress. The progress phase is designed to effectively minimize the loss of a specified task, i.e., $\mathcal{L}^{progress} = -\mathbb{E}_\pi \left[\sum_{n=0}^{\infty} \gamma^n r_{t+n}\right]$, which is equivalent to the RL objective $\max_\pi \mathbb{E}_\pi \left[\sum_{n=0}^{\infty} \gamma^n r_{t+n}\right]$, where r is the reward and γ is the discount factor. As in Schwarz et al. [13], we used a distributed variant of the actor-critic architecture (A2C) to learn both the policy $\pi(a_t|s_t;\theta_1)$ and the value function $V(s_t;\theta_2)$, from raw pixels, within both phases. The policy and value function (both referred to as *active column*) share a convolutional encoder, similar to the approach described by Mnih et al. [37].
Compress. The compress phase incorporates a variety of methods, making the implementation and fine-tuning of hyperparameters the most time-intensive aspect of this process. Unlike the progress phase, during which the primary focus is on one task, the compress phase is crucial for retaining knowledge as tasks are encountered sequentially. After acquiring knowledge for a designated number of timesteps during the progress phase, the most recent policy (the active column) of the current task is distilled into the knowledge base network (the same copy of the network architecture was used as in the progress phase) by minimizing the following distillation loss with respect to the parameters θ_{kb} of the knowledge base:

$$\mathcal{L}^{Distill}(\theta_{kb}) = \mathbb{E}\left[D_{\mathrm{KL}}\left(\pi_k(\cdot|x;\theta_{active}) \parallel \pi_{kb}(\cdot|x;\theta_{kb})\right)\right] + \frac{\lambda}{2}\gamma F_{k-1}\left\|\theta_{kb} - \theta_{kb}^{*(k-1)}\right\|_2^2 \quad \text{(A1)}$$

where x is the input and $\pi_k(\cdot|x;\theta_{active})$, $\pi_{kb}(\cdot|x;\theta_{kb})$ are the policies of the active column (after learning on task k) and the knowledge base, respectively. F_{k-1} is the diagonal Fisher Information Matrix (FIM) of the previous tasks and $\gamma > 0$ is the forgetting constant, introducing gracefully forgetting old knowledge (mind the overloaded notation γ). λ is the importance of the penalty term. $\theta_{kb}^{*(k-1)}$ are the optimal parameters of the previous tasks. The loss function $\mathcal{L}^{Distill}(\theta_{kb})$ is then estimated by stochastic gradient descent, finding $\theta_{kb}^* = \arg\min_{\theta_{kb}} \mathcal{L}^{Distill}(\theta_{kb})$. This optimization is referred to in Schwarz et al. [13] as online Elastic Weight Consolidation or Online EWC in short. It is important that the penalty can only be applied after the first compress phase. The knowledge base not only acts as a repository of cumulative knowledge but also enables the active column to build upon this consolidated information. The integrated knowledge serves as a foundation for the model to further refine and optimize its learning processes. This iterative learning allows for enhancing the model's performance on subsequent tasks.

Before any subsequent compress phase starts, the retainment of the knowledge base's current parameters θ_{kb}^* is necessary. For doing this, it is important that after the very first compress phase, the diagonal of the Fisher information matrix gets estimated with respect to the latest compressed optimal parameters θ_{kb}^* of the knowledge base network. As the A2C is an on-policy algorithm, in order to calculate the Fisher estimate w.r.t. θ_{kb}^*, we need to generate samples based on the policy $\pi_{kb}(\cdot|x;\theta_{kb}^*)$ to have an accurate estimation of the diagonal of the Fisher information. Estimating the

Fisher information is as follows:

$$F(\theta_{kb}^{*}) = \frac{1}{N}\sum_{t=1}^{N}\left(\frac{\partial(\log(\pi_{kb}(a_t|s_t;\theta_{kb}^{*}))\cdot A_t)}{\partial\theta_{kb}^{*}}\right)^2 \tag{A2}$$

where N is the number of samples generated by the policy $\pi_{kb}(\cdot|x;\theta_{kb}^{*})$. The algorithmic procedure is outlined in Algorithm 1.

Algorithm 1 Distillation + Online EWC Algorithm

Input: Tasks $\{1, 2, \ldots, T\}$, forgetting factor γ, regularization hyperparameter λ

Initialize: Distill (train) Task 1 to obtain $\theta_{kb}^{*(1)} = \arg\min_{\theta_{kb}} \mathcal{L}_1^{Distill}(\theta_{kb})$

for each task $t > 1$ **do**

  Compute FIM: $F_{t-1} = \mathbb{E}_{s_{t-1}\sim P_{t-1}(\cdot)}\left[\left(\frac{\partial(\log(\pi_{kb}(a_{t-1}|s_{t-1};\theta_{kb}^{*(t-1)}))\cdot A_{t-1})}{\partial\theta_{kb}^{*(t-1)}}\right)^2\right]$

  Online FIM Update: $F_{t-1} = \gamma F_{t-2} + F_{t-1}$

  Distillation Loss: $\mathcal{L}_t^{Distill}(\theta_{kb}) = \mathcal{L}_t(\theta_{kb}) + \frac{\lambda}{2}F_{t-1}\left\|\theta_{kb} - \theta_{kb}^{*(t-1)}\right\|_2^2$

  Update Parameters: $\theta_{kb}^{*(t)} = \arg\min_{\theta_{kb}} \mathcal{L}_t^{Distill}(\theta_{kb})$

end for

Architecture. An identical architecture is used within both the progress and compress phases. The active column consists of one policy $\pi_{active}(a_t|s_t;\theta_{active}^1)$ and a value function $V_{active}(s_t;\theta_{active}^2)$ which share a convolutional encoder $\phi_{active}(\cdot;\theta_{active}^3)$. This encoder is represented by a convolutional neural network (CNN), following the approach outlined by Mnih et al. [37]. Let s_t represent the sequence of observations at timestep t from the environment, where $\phi_{active}(s_t)$ indicates the encoded visual features used for subsequent policy learning. The same principle applies to the knowledge base network, with the only difference being the change in the subscript to *kb*. It is important to note that the active column incorporates lateral connections from the knowledge base. This is done through the function $f(\cdot)$ represented by a neural network consisting of convolutional layers and a multi-layer perceptron with one hidden layer and designed to process the intermediate outputs from the knowledge base. These intermediate outputs are derived from $\phi_{kb}(s_t)$, $\pi_{kb}(a_t|s_t;\theta_{kb}^1)$, and $V_{kb}(s_t;\theta_{kb}^2)$. Initially, during the training of the first task for a specified number of timesteps, the active column does not utilize any lateral connections, keeping the knowledge base inactive. However, after the compress phase, the lateral connections are activated, utilizing prior knowledge to optimize both the active column and the lateral connections in the progress phase of the next task.

Learning in the Progress and Compress Framework. Algorithm 2 represents the approach of Schwarz et al. [13] in the RL domain, unifying all arguments mentioned in the previous sections. In every iteration, a vectorized environment is constructed corresponding to each task.

Algorithm 2 Vanilla Progress and Compress Algorithm

Initialize: policy networks π_{active}, π_{kb}; encoders ϕ_{active}, ϕ_{kb}; tasks $\mathcal{T}$; timesteps $T_{active}, T_{kb}, T_{Fisher}$; buffer U; encoder f; visits $\mathcal{V}$

for each visit v in $\mathcal{V}$ **do**
    for each task k in $\mathcal{T}$ **do**
        Set up environment E^k
        Unfreeze $\pi_{active}, \phi_{active}, f$; Freeze π_{kb}, ϕ_{kb}
        for $t = 1$ to T_{active} **do** ▷ Progress Phase
            Observe $s_t \sim E^k$, $\phi = \phi_{active}(s_t)$, $a_t \sim \pi_{active}(f(\phi))$
            $r_{t+1}, s_{t+1} \sim P^{E^k}(s_t, a_t)$, $U \leftarrow U \cup \{(r_{t+1}, s_t, s_{t+1}, a_t)\}$
            if $t \mod \text{size}(U) = 0$ **then**
                Evaluate actions based on rollout U
                Update θ_{active} using SGD; Clear U ▷ the loss $\mathcal{L}^{progress}(\theta_{active})$
            end if
        end for
        Switch modes: Freeze $\pi_{active}, \phi_{active}$; Unfreeze π_{kb}, ϕ_{kb}
        for $t = 1$ to T_{kb} **do** ▷ Compress Phase
            Observe $s_t \sim E^k$, $\phi = \phi_{kb}(s_t)$, $a_t \sim \pi_{kb}(\phi)$
            $s_{t+1} \sim P^{E^k}(s_t, a_t)$, $U \leftarrow U \cup \{(s_t, s_{t+1}, a_t)\}$
            if $t \mod \text{size}(U) = 0$ **then**
                if $k < 2$ **then**
                    Update θ_{kb} with SGD on $D_{\text{KL}}(\pi_{active}(\cdot|s_{t:\text{size}(U)}) \parallel \pi_{kb}(\cdot|s_{t:\text{size}(U)}))$
                else
                    Update θ_{kb} with SGD on $D_{\text{KL}}(\pi_{active}(\cdot|s_{t:\text{size}(U)}) \parallel \pi_{kb}(\cdot|s_{t:\text{size}(U)}))$ $+\frac{\lambda}{2} F_{k-1} \left\| \theta_{kb} - \theta_{kb}^{*(k-1)} \right\|_2^2$
                end if
                Clear U
            end if
        end for
        for $t = 1$ to T_{Fisher} **do**
            Observe $s_t \sim E^k$, $\phi = \phi_{kb}(s_t)$, $a_t \sim \pi_{kb}(\phi)$, $s_{t+1} \sim P^{E^k}(s_t, a_t)$
            $U \leftarrow U \cup \{(s_t, s_{t+1}, a_t)\}$
            if $t \mod \text{size}(U) = 0$ **then**
                Estimate Fisher information based on U; Clear U
            end if
        end for
        Update Fisher information for θ_{kb}^*; Reinitialize θ_{active} ▷ see Equation A2
    end for
end for

Appendix B Architecture Details

B.1 A2C Hyperparameters

The following hyperparameters (see Table B1) specify both the active column and knowledge base networks, which are used in both progress and compress phases for representing the actor (policy) and critic (value function).

Table B1: Neural network architecture of the active column and knowledge base

Order	Layer Type	Activation	Size	Filter Size	Filter Stride
1	Convolution	ReLU	32	[8 × 8]	[4 × 4]
2	Convolution	ReLU	64	[4 × 4]	[2 × 2]
3	Convolution	ReLU	32	[3 × 3]	[1 × 1]
4	Flatten	-	-	-	-
5	Fully-Connected	ReLU	512 Neurons	-	-
6	Critic	Linear	1 Neuron	-	-
7	Actor(Policy)	Linear	4 Neurons	-	-

B.2 Forward Model Hyperparameters

The following hyperparameters (see Table B2) are utilized to assemble the forward model incorporated in the implementation. In this process, the action is one-hot encoded and subsequently merged with an encoded state feature representation.

Table B2: Neural network architecture of the forward model

Order	Layer Type	Activation	Size	Filter Size	Filter Stride
1	Convolution	ReLU	32	[3 × 3]	[2 × 2]
2	Convolution	ReLU	32	[3 × 3]	[2 × 2]
3	Convolution	ReLU	32	[3 × 3]	[2 × 2]
4	Convolution	ReLU	32	[3 × 3]	[2 × 2]
5	Convolution	ReLU	32	[3 × 3]	[2 × 2]
6	Fully-Connected	ReLU	256 Neurons	-	-
7	Fully-Connected	Linear	288 Neurons	-	-

B.3 Hyperparameters of the Experiments

All variations in timesteps across experiments are explicitly stated. One important parameter is the “num-samples-drawn-in-task-agnostic-phase” (see Table B3). This represents a procedure in the task-agnostic phase where games are randomly selected and trained for a specific duration set by “num-env-steps-agnostic”. Then, the compress phase is executed for “num-env-steps-compress-agnostic”. This process is repeated 30 times, where 30 samples are uniformly drawn from the Meta-Environment. The Meta-Environment includes Pong (P), SpaceInvaders (S), and BeamRider (B). The RMSprop optimizer was consistently used in all experiments.

Table B3: Hyperparameter values in the Progress & Compress baseline, TAPD, Progressive Nets, and Online EWC of the task-agnostic phase

**Parameter**	**Baseline**	**Task-Agnostic Policy Distillation (TAPD)**	**Progressive Nets**	**Online EWC**
agnostic-phase	-	True	-	-
batch-size-fisher (Fisher information estimation)	32			
eval-steps	10^5			
ewc-lambda	2			
ewc-gamma	0.3			
gamma (Discount factor for rewards)	0.99			
ewc-start	15×10^4			
entropy-coef (Entropy term coefficient)	0.01			
lr (Learning rate)	7×10^{-4}			
eps (RMSprop optimizer epsilon)	1×10^{-5}			
alpha (RMSprop optimizer alpha)	0.99			
num-env-steps-agnostic	-	3×10^5	-	-
num-env-steps-compress (T_{kb})	3×10^5		-	-
num-env-steps-agnostic-compress	-	3×10^5	-	-
num-env-steps-progress (T_{active})	2.5×10^6			
num-processes	10			
num-visits	3			
value-loss-coef	0.5			
max-grad-norm	0.5			
num-steps-fisher (T_{Fisher}) [11]	100			
num-steps (Rollout size)	20			
num-samples-drawn-in-task-agnostic-phase	-	25	-	-
Tasks in task-agnostic phase	-	S, B	-	-
Tasks in progress and compress phases / Training phase	Pong (P), SpaceInvaders (S), BeamRider (B), DemonAttack (D), AirRaid (A)			
